# Impact of the COVID-19 Pandemic on Health Check-ups before and after the COVID-19 Downgrade and Employees’ Mental Health at Health Check-up Facilities: The Third Nationwide Survey of Healthcare Facilities in Japan Society of Ningen Dock and Preventive Medical Care

**DOI:** 10.31662/jmaj.2024-0337

**Published:** 2025-06-27

**Authors:** Satoko Yamaguchi, Tomofumi Atarashi, Akira Okada, Reiko Inoue, Shigeru Nasu, Toshimasa Yamauchi, Yasuji Arase, Takao Aizawa, Masaomi Nangaku, Takashi Kadowaki

**Affiliations:** 1 Department of Prevention of Lifestyle-Related Diseases, Graduate School of Medicine, The University of Tokyo, Tokyo, Japan; 2 Japan Society of Ningen Dock and Preventive Medical Care, Tokyo, Japan; 3 Center for Preventive Medicine, The Jikei University School of Medicine, Tokyo, Japan; 4 Medical Check-up Center, JA Hokkaido P.W.F.A.C. Obihiro-Kosei General Hospital, Obihiro, Japan; 5 Hakuaikai Hospital, Fukuoka, Japan; 6 Department of Diabetes and Metabolic Diseases, Graduate School of Medicine, The University of Tokyo, Tokyo, Japan; 7 Health management center, Toranomon Hospital, Tokyo, Japan; 8 Aizawa Hospital, Nagano, Japan; 9 Division of Nephrology and Endocrinology, Graduate School of Medicine, The University of Tokyo, Tokyo, Japan; 10 Toranomon Hospital, Tokyo, Japan

**Keywords:** COVID-19 pandemic, health check-up, cancer screening, mental health, attrition

## Abstract

**Introduction:**

Preventive programs, including cancer and diabetes screenings, were disrupted globally by the coronavirus disease 2019 (COVID-19) pandemic and had not returned to pre-pandemic levels even in 2021-2022. In Japan, COVID-19 was downgraded to the lowest-risk category under the Infectious Diseases Control Law in May 2023. However, whether participation in health check-ups recovered after this downgrade remains unclear. Additionally, understanding the impact of the pandemic on employees’ mental health and attrition is crucial for maintaining services in future pandemics. To address these issues, we conducted a nationwide survey.

**Methods:**

A questionnaire survey was conducted between December 16, 2023, and February 21, 2024, targeting member facilities of Japan Society of Ningen Dock and Preventive Medical Care. The questionnaires covered COVID-19-related rules before and after the downgrade, the negative impact on employees’ mental health, and whether employee resignations increased compared to the pre-pandemic period. Participants also provided data on the number of health check-up examinees from 2019 to 2023.

**Results:**

We emailed 1,381 facilities, of which 856 responded (response rate: 62.0%). An additional 124 facilities responded via the Society’s website, yielding a total of 980 participants. While cancer screenings by local governments returned to pre-pandemic levels in 2023, gastric cancer screenings remained low. Nearly 30% of facilities reported a negative impact of the pandemic on employees’ mental health, which was associated with factors such as being annexed to hospitals and employees’ complaints about strict COVID-19-related rules. This negative impact was strongly linked to increased employee resignations.

**Conclusions:**

While overall cancer screening rates recovered, gastric cancer screenings remained below pre-pandemic levels. Furthermore, the negative impact on employees’ mental health was significantly associated with increased attrition, highlighting the need for mental health support for those engaged in preventive medicine to maintain services during future pandemics.

## Introduction

In 2020, the coronavirus disease 2019 (COVID-19) pandemic caused significant worldwide disruptions to preventive programs, including cancer and diabetes screenings ^(1), (2)^, raising concerns regarding delayed diagnosis and treatment ^(3), (4)^. Reports suggested that cancer screenings remained below pre-pandemic levels even in 2021 ^(5), (6)^. Furthermore, it was reported that cervical cancer screening rates were lower in 2022 compared to the pre-pandemic level in the United States ^(7)^, and we also reported that the number of people undergoing non-mandatory cancer screenings had not recovered in 2022 ^(8)^.

Japan has developed nationwide health check-up programs. Employers are obliged to provide annual mandatory check-ups for full-time employees under the Industrial Safety and Health Act ^(9)^, and it is mandatory for employees to undergo them. All insurers are obliged to provide annual Specific Health Check-ups for all people aged 40-74 years ^(10)^. Additionally, local governments are required to make efforts to provide cancer screenings, but it is not mandatory for individuals to undergo them ^(11)^. We previously conducted two nationwide questionnaire surveys to investigate the impact of the pandemic on health check-ups, covering the periods from 2020 to 2021 and from 2020 to 2022 ^(8), (12)^. Although the number of examinees undergoing mandatory check-ups recovered in 2021 from the decline in 2020, the number of those undergoing non-mandatory check-ups remained low in 2021, and this tendency persisted into 2022. Moreover, employees in health check-up facilities were subject to strict COVID-19-related rules, leading to considerable sacrifices in their personal lives, a trend that was particularly evident in facilities annexed to hospitals.

In Japan, COVID-19 was downgraded on May 8, 2023, from the same category as novel influenza (equivalent to Category II) to a Category V infectious disease, the lowest-risk category under the Infectious Diseases Control Law, which is the same as that of seasonal influenza ^(13)^. Following the downgrade, non-pharmaceutical interventions (NPIs), such as screening and isolation of sick individuals, quarantine of exposed individuals, social distancing, and hand washing were lifted. Prior to this downgrade, mask-wearing guidelines were eased in March 2023; however, recommendations to wear masks in hospital or care home settings continued to protect those vulnerable to COVID-19 infection.

While evaluating the impact of the pandemic on examinees’ participation in health check-ups is important to prepare for future pandemics, the impact on employees working in health check-up facilities must also be considered. Preventing employee attrition is crucial to maintaining services, especially during crises such as pandemics and the subsequent recovery periods. Recent studies suggest that mental health has been a key factor contributing to attrition among healthcare workers since the COVID-19 pandemic ^(14), (15)^. Therefore, understanding the impact of the pandemic on employees’ mental health and the factors associated with this impact is crucial for sustaining healthcare services during future pandemics. Although the impact of the pandemic on healthcare workers, particularly regarding mental health and attrition, has been widely studied, its impact on those engaged in preventive programs remains unknown. A higher prevalence of mental health problems among healthcare workers was reported during the pandemic ^(16), (17)^, leading to increased workforce attrition. In Japan, some facilities are annexed to hospitals, with check-up facilities and hospitals typically located on the same premises. These facilities offer the advantage of immediate access to hospital resources if abnormalities are detected during health check-ups. Although employees of health check-up facilities are generally not working in high-risk units, which is a major risk factor for deteriorating mental health ^(16), (18)^, approximately half of the facilities annexed to hospitals reported that their employees had to contribute to COVID-19-related care in our previous survey ^(8)^. Moreover, COVID-19-related restrictions were reported to have adverse effects on mental health among the general population ^(19), (20)^, and employees of health check-up facilities were likely subject to more stringent COVID-19-related restrictions compared to others, even after the COVID-19 downgrade. Therefore, the mental health of employees in health check-up facilities was likely significantly impacted by the pandemic, even though they were not primarily involved in COVID-19-related care. In particular, facilities annexed to hospitals were more likely to provide COVID-19-related care and impose stricter COVID-19-related rules on their employees ^(8)^. These factors may have contributed to increased stress and a higher risk of mental health issues among employees.

This study, based on the third nationwide survey of healthcare facilities, examines the impact of the pandemic up to 2023, focusing on whether participation in health check-ups returned to pre-pandemic levels after NPIs were lifted. Additionally, it evaluates the pandemic’s effects on employees of health check-up facilities, particularly regarding mental health and attrition.

## Materials and Methods

### Questionnaire survey

A questionnaire survey was administered, as previously described ^(8), (12)^. Briefly, healthcare facilities that were members of Japan Society of Ningen Dock and Preventive Medical Care and could respond via email were considered eligible. Japan Society of Ningen Dock and Preventive Medical Care had 1,813 member facilities at the time of the survey, including hospitals and clinics across Japan ^(21)^. Emails, including links to web-based questionnaires and fillable forms, were sent to eligible facilities on December 16, 2023. In addition, these links were made available on the Society’s homepage, so that member facilities that did not receive the emails could also respond.

The web-based questionnaires included questions on the type of facilities, the number of employees engaged in health check-ups per day, whether the facilities were providing inpatient or outpatient care for patients with COVID-19, precautions taken against COVID-19 to perform in-facility check-ups, and COVID-19-related rules in place during the year before and the period after the COVID-19 downgrade, whether the COVID-19 pandemic had a negative financial impact on the management of health check-ups, whether employee resignations increased or new recruits decreased compared to the pre-pandemic period, whether the pandemic had a negative impact on employees’ mental health, whether employees complained that COVID-19-related rules were too strict, and whether there were employees experiencing difficulty working due to post-COVID-19 condition, commonly known as long COVID. Facilities annexed to hospitals were asked about the number of beds and whether they provided emergency care. Additionally, to assess the degree of check-up facilities’ independence from annexed hospitals, facilities annexed to hospitals were asked whether personnel recruitment for check-up facilities was independent of recruitment for the annexed hospitals, and whether there was personnel transfer between the check-up facility and the annexed hospital.

Participants were also asked to fill in a form about the number of examinees who underwent health check-ups between 2019 and 2023, according to the type of check-ups. Responses were collected by February 21, 2024.

### Outcome measures

To evaluate whether the number of examinees undergoing health check-ups has returned to pre-pandemic levels, the total number of examinees undergoing check-ups in 2020, 2021, 2022, and 2023 was compared with that in 2019 (the pre-COVID-19 year) in 430 facilities that consistently provided data annually between 2019 and 2023. To evaluate the impact of the pandemic on employees’ mental health, facilities were asked whether it had a negative impact, and associated factors were identified as described below. Furthermore, the association between this negative impact and increased employee resignations was examined.

### Statistical analysis

To assess the pandemic’s impact and the characteristics associated with its negative effect on employees’ mental health, facility characteristics were compared between hospital-annexed and non-annexed facilities and between facilities with and without the negative impact, using the χ^2^ test or Fisher’s exact test. To evaluate potential selection bias, facility characteristics were compared between participating and non-participating facilities using the χ^2^ test.

Precautions taken against COVID-19 to perform in-facility check-ups and COVID-19-related rules were compared before and after the COVID-19 downgrade using McNemar’s test.

To identify factors associated with the negative impact on employees’ mental health, multivariate logistic regression analyses were performed, with the negative impact on mental health as the dependent variable and facility characteristics, including type, scale, location, and COVID-19-related rules, as independent variables. The analyses were conducted after excluding one variable from each pair of variables with an absolute Spearman’s correlation coefficient greater than 0.6.

All statistical tests were two-sided, and P values < 0.05 were considered significant. All analyses were performed using R v4.2.3. (R Foundation for Statistical Computing, Vienna, Austria).

### IRB approval

This study was approved by the institutional review board of the Graduate School of Medicine at the University of Tokyo (2018030NI) and was performed in accordance with the relevant guidelines and regulations (the Declaration of Helsinki). The requirement for informed consent was waived because the facility-based survey did not contain any personal information.

## Results

Emails requesting participation in the survey were sent to 1,381 facilities, of which 856 responded (response rate 62.0%). In addition, 124 facilities that did not receive emails participated in the survey through the Japan Society of Ningen Dock and Preventive Medical Care website (Supplementary Figure 1). Overall, 980 facilities participated; 461 responded to both the web-based questionnaire and the fillable form, 498 responded to the web-based questionnaire only, and 21 responded to the fillable form only. To assess potential selection bias, the characteristics of facilities that participated in this study were compared with those that did not (Supplementary Table 1). The results suggested that facilities in the Hokkaido area may be underrepresented, while no differences were observed regarding urban location or public institution status.

### The number of examinees undergoing health check-ups in 2023

**Figure 1.**
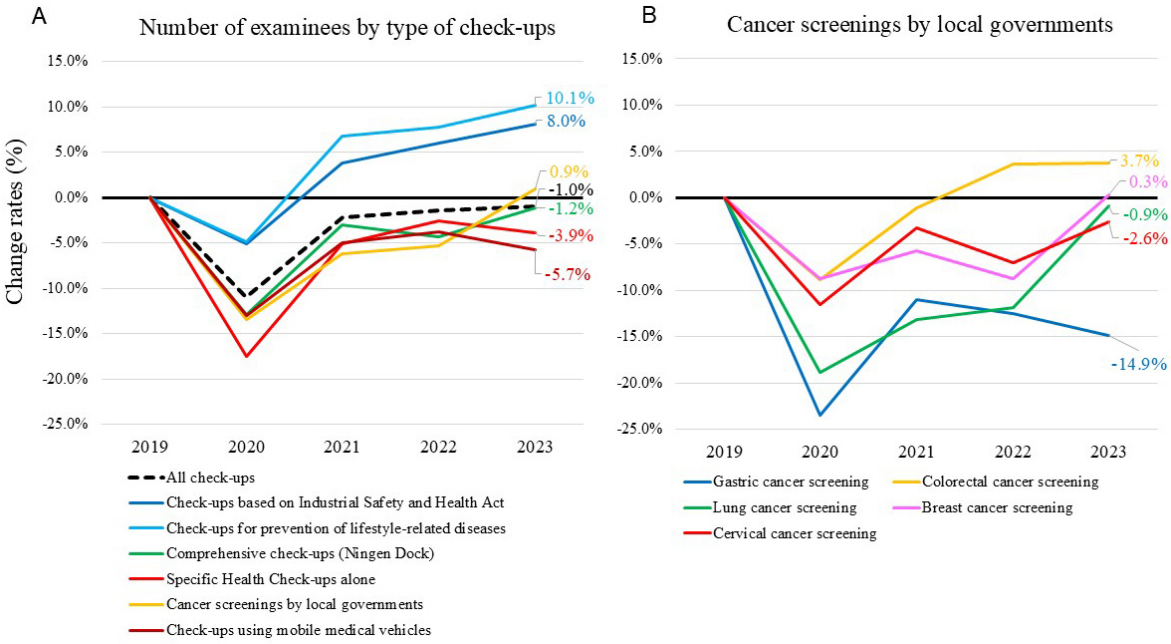
A: Change rates in the number of examinees undergoing different types of check-ups in 430 facilities, compared to 2019. B: Change rates in the number of examinees undergoing “Cancer screenings by local governments” by type of screening, compared to 2019.

We evaluated whether the number of examinees undergoing health check-ups has returned to the pre-pandemic levels in 2023. The total number of examinees undergoing check-ups at 430 facilities in each year between 2020 and 2023 was compared with that in 2019. As we previously reported, while the number of examinees undergoing “Check-ups based on Industrial Safety and Health Act” and “Check-ups for prevention of lifestyle-related diseases,” both primarily undertaken by full-time employees required to have annual health check-ups, recovered in 2021 from the decline in 2020, the number of examinees undergoing non-mandatory “Specific Health Check-ups alone” and “Cancer screenings by local governments” had not fully recovered even in 2022 ^(8)^. The change rates in the number of examinees undergoing non-mandatory “Specific Health Check-ups alone” and “Cancer screenings by local governments” in 2023 were −3.9% and 0.9%, respectively (Figure 1A and Supplementary Table 2). Among “Cancer screenings by local governments,” change rates for gastric, colorectal, lung, breast, and cervical cancer screenings were −14.9%, 3.7%, −0.9%, 0.3%, and −2.6%, respectively (Figure 1B).

### Characteristics of the participating facilities by annexation to hospitals

We previously reported that the pandemic had a greater impact on health check-up facilities annexed to hospitals than on those not annexed. These facilities were more likely to provide COVID-19-related care, impose stricter COVID-19-related rules on their employees, and experience a negative financial impact ^(8)^. Therefore, we compared the characteristics between facilities annexed to hospitals and facilities not annexed to hospitals (Table 1). Among the 959 facilities that responded to the web-based questionnaire, 569 (59.3%) were annexed to hospitals, while 390 (40.7%) were not. Compared to facilities not annexed to hospitals, those annexed to hospitals were less likely to be in ordinance-designated cities or special wards in Tokyo, more likely to be public, have fewer employees, and provide inpatient and outpatient care for patients with COVID-19, consistent with the previous surveys ^(8), (12)^. Among the 569 facilities annexed to hospitals, personnel recruitment for check-up facilities was independent of recruitment for annexed hospitals in 243 (42.7%) facilities. Personnel transfers between check-up facilities and annexed hospitals occurred in 447 (78.6%) facilities, suggesting that the pandemic situation in the hospitals is relevant to employees in most check-up facilities annexed to hospitals.

**Table 1.** Characteristics of the Facilities That Participated in the Web-based Questionnaire by Facility Type.

		Total (n = 959)	Not annexed to hospitals (n = 390)	Annexed to hospitals (n = 569)	P value^a^
Area^b^	Kanto	331 (34.5)	154 (39.5)	177 (31.1)	0.007
	Hokkaido	19 (2.0)	8 (2.1)	11 (1.9)	
	Tohoku	54 (5.6)	23 (5.9)	31 (5.4)	
	Chubu	179 (18.7)	60 (15.4)	119 (20.9)	
	Kansai	188 (19.6)	87 (22.3)	101 (17.8)	
	Chugoku	73 (7.6)	26 (6.7)	47 (8.3)	
	Shikoku	24 (2.5)	9 (2.3)	15 (2.6)	
	Kyushu	82 (8.6)	21 (5.4)	61 (10.7)	
	Okinawa	9 (0.9)	2 (0.5)	7 (1.2)	
Location of facilities	Ordinance-designated cities/special wards in Tokyo	379 (39.5)	219 (56.2)	160 (28.1)	<0.001
Public or private institutions	Public	182 (19.0)	15 (3.8)	167 (29.3)	<0.001
Number of employees	1-10	219 (22.8)	73 (18.7)	146 (25.7)	<0.001
	11-20	249 (26.0)	69 (17.7)	180 (31.6)	
	21-40	270 (28.2)	92 (23.6)	178 (31.3)	
	>40	221 (23.0)	156 (40.0)	65 (11.4)	
Providing inpatient care for patients with COVID-19	Yes	482 (50.3)	4 (1.0)	478 (84.0)	<0.001
Providing outpatient care for patients with COVID-19	Yes	648 (67.6)	125 (32.1)	523 (91.9)	<0.001
Number of beds	<100	N/A	N/A	72 (12.7)	N/A
	100-299	N/A	N/A	282 (49.6)	
	300-499	N/A	N/A	146 (25.7)	
	≥500	N/A	N/A	63 (11.1)	
	Unanswered	N/A	N/A	6 (1.1)	
Emergency care	No emergency care	N/A	N/A	71 (12.5)	N/A
	Primary	N/A	N/A	100 (17.6)	
	Secondary or tertiary	N/A	N/A	391 (68.7)	
	Unanswered	N/A	N/A	7 (1.2)	
Personnel recruitment for check-up facilities is independent of recruitment for annexed hospitals	No	N/A	N/A	321 (56.4)	N/A
	Yes	N/A	N/A	243 (42.7)	
	Unanswered	N/A	N/A	5 (0.9)	
Personnel transfer between the check-up facility and the annexed hospital	No	N/A	N/A	118 (20.7)	N/A
	Yes	N/A	N/A	447 (78.6)	
	Unanswered	N/A	N/A	4 (0.7)	
Negative financial impact in 2023	Yes	235 (24.5)	83 (21.3)	152 (26.7)	0.065
Employee resignations compared to the pre-pandemic period	Increased	104 (10.8)	20 (5.1)	84 (14.8)	<0.001
New recruits in 2023 compared to 2019	Decreased	123 (12.8)	29 (7.4)	94 (16.5)	<0.001
Employees complain that COVID-19-related rules are too strict	Yes	73 (7.6)	17 (4.4)	56 (9.8)	0.003
Negative impact on the mental health of check-up facility employees	Yes	280 (29.2)	90 (23.1)	190 (33.4)	<0.001
Some employees have difficulty working due to long COVID	Yes	74 (7.7)	27 (6.9)	47 (8.3)	0.523
Organizational social gathering involving meals held in FY2019/FY2023	FY2019: no FY2023: no	224 (23.4)	96 (24.6)	128 (22.5)	0.069
	FY2019: no FY2023: yes	227 (23.7)	91 (23.3)	136 (23.9)	
	FY2019: yes FY2023: no	273 (28.5)	95 (24.4)	178 (31.3)	
	FY2019: yes FY2023: yes	235 (24.5)	108 (27.7)	127 (22.3)	

^a^P value comparing facilities annexed to hospitals and those not annexed using the χ^2^ test. ^b^Areas of Japan: Japan comprises the four main islands of Hokkaido, Honshu, Shikoku, and Kyushu. Honshu, the largest island, is divided into Kanto (which includes the Greater Tokyo area), Tohoku, Chubu, Kansai, and Chugoku regions. Okinawa is the southernmost prefecture that includes the fifth-largest island. FY: fiscal year; N/A: not applicable.

We evaluated whether the pandemic had a greater negative impact on facilities annexed to hospitals in terms of employees’ mental health and attrition. A negative impact on employees’ mental health was reported by 29.2% of all facilities, and indeed, facilities annexed to hospitals were more likely to report a negative impact on employees’ mental health compared to those not annexed to hospitals. Furthermore, facilities annexed to hospitals were more likely to report an increase in employee resignations and a decrease in new recruits compared to the pre-pandemic period. They were also more likely to report complaints from employees about COVID-19-related rules being too strict.

### Precautions against COVID-19 before and after the COVID-19 downgrade

**Figure 2.**
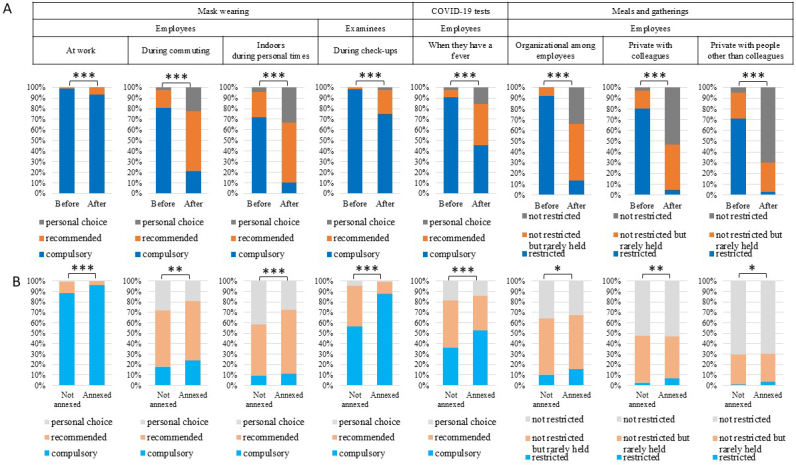
A: COVID-19-related rules during the year before and the period after the COVID-19 downgrade were compared using McNemar’s test (n = 959). B: COVID-19-related rules after the COVID-19 downgrade were compared between facilities annexed to hospitals (n = 569) and those not annexed to hospitals (n = 390) using the χ^2^ test. * P < 0.05, ** P < 0.01, *** P < 0.001.

To evaluate the impact of the COVID-19 downgrade on precautions taken for in-facility check-ups and COVID-19-related rules, we compared the precautions and the COVID-19-related rules implemented during the year before and after the downgrade. We also evaluated whether employees in facilities annexed to hospitals were subject to stricter COVID-19-related rules than those in facilities not annexed to hospitals, even after the downgrade. The implementation rates of precautions declined (Supplementary Table 3 and Supplementary Figure 2), and COVID-19-related rules were generally relaxed after the COVID-19 downgrade (Table 2 and Figure 2); however, wearing masks at work was compulsory in more than 90% of the facilities even after the downgrade. Organizational social gatherings among employees involving meals and private meals and gatherings with people other than colleagues were restricted in over 90% and 70% of facilities, respectively, during the year before the downgrade. After the downgrade, restrictions were in place in 13.3% and 2.7% of facilities, respectively. However, 52.6% and 27.4% of the facilities reported that while such gatherings were not restricted, they were rarely held at the time of the survey. Facilities annexed to hospitals imposed stricter rules than those not annexed after the downgrade (Table 2 and Figure 2).

**Table 2.** COVID-19-related Rules before and after the Downgrade.

		Before vs after COVID-19 downgrade			After COVID-19 downgrade		
		Before (n = 959)	After (n = 959)	P value^a^	Not annexed to hospitals (n = 390)	Annexed to hospitals (n = 569)	P value^b^
Employees							
Wearing masks at work	Compulsory	951 (99.2)	893 (93.1)	<0.001	345 (88.5)	548 (96.3)	<0.001
	Recommended	8 (0.8)	62 (6.5)		43 (11.0)	19 (3.3)	
	Personal choice	0 (0.0)	4 (0.4)		2 (0.5)	2 (0.4)	
Wearing masks during commuting	Compulsory	771 (80.4)	203 (21.2)	<0.001	68 (17.4)	135 (23.7)	0.002
	Recommended	165 (17.2)	538 (56.1)		213 (54.6)	325 (57.1)	
	Personal choice	23 (2.4)	218 (22.7)		109 (27.9)	109 (19.2)	
Wearing masks indoors during personal time	Compulsory	690 (71.9)	101 (10.5)	<0.001	37 (9.5)	64 (11.2)	<0.001
	Recommended	230 (24.0)	539 (56.2)		191 (49.0)	348 (61.2)	
	Personal choice	39 (4.1)	319 (33.3)		162 (41.5)	157 (27.6)	
Taking COVID-19 tests when having a fever	Compulsory	868 (90.5)	438 (45.7)	<0.001	140 (35.9)	298 (52.4)	<0.001
	Recommended	68 (7.1)	370 (38.6)		178 (45.6)	192 (33.7)	
	Personal choice	23 (2.4)	151 (15.7)		72 (18.5)	79 (13.9)	
Number of days employees are not allowed to work after a COVID-19 infection	No restrictions	N/A	N/A	N/A	12 (3.1)	6 (1.1)	<0.001
	5 days	N/A	N/A		321 (82.3)	396 (69.6)	
	7 days	N/A	N/A		48 (12.3)	137 (24.1)	
	≥10 days	N/A	N/A		4 (1.0)	22 (3.9)	
	Others	N/A	N/A		5 (1.3)	8 (1.4)	
When family members are infected with COVID-19	Employees are not allowed to work for a certain period	901 (94.0)	210 (21.9)	<0.001	70 (17.9)	140 (24.6)	0.045
	Employees are allowed to work if they are not sick	57 (5.9)	728 (75.9)		310 (79.5)	418 (73.5)	
	No restrictions	1 (0.1)	21 (2.2)		10 (2.6)	11 (1.9)	
Organizational social gathering among employees involving meals (hospital-wide year-end party, department-wide farewell party)	Restricted	884 (92.2)	128 (13.3)	<0.001	38 (9.7)	90 (15.8)	0.024
	Not restricted but rarely held	70 (7.3)	504 (52.6)		212 (54.4)	292 (51.3)	
	Not restricted	5 (0.5)	327 (34.1)		140 (35.9)	187 (32.9)	
Private meals and gatherings with colleagues	Restricted	770 (80.3)	48 (5.0)	<0.001	10 (2.6)	38 (6.7)	0.010
	Not restricted but rarely held	163 (17.0)	404 (42.1)		176 (45.1)	228 (40.1)	
	Not restricted	26 (2.7)	507 (52.9)		204 (52.3)	303 (53.3)	
Private meals and gatherings with people other than colleagues	Restricted	682 (71.1)	26 (2.7)	<0.001	4 (1.0)	22 (3.9)	0.024
	Not restricted but rarely held	228 (23.8)	263 (27.4)		113 (29.0)	150 (26.4)	
	Not restricted	49 (5.1)	670 (69.9)		273 (70.0)	397 (69.8)	
Examinees							
Wearing masks When undergoing check-ups	Compulsory	940 (98.0)	719 (75.0)	<0.001	221 (56.7)	498 (87.5)	<0.001
	Recommended	18 (1.9)	215 (22.4)		150 (38.5)	65 (11.4)	
	Personal choice	1 (0.1)	25 (2.6)		19 (4.9)	6 (1.1)	
Undergoing check-ups after having a fever	Not allowed for a certain time	842 (87.8)	482 (50.3)	<0.001	157 (40.3)	325 (57.1)	<0.001
	Allowed if the COVID-19 test is negative	99 (10.3)	216 (22.5)		98 (25.1)	118 (20.7)	
	Allowed if the fever has broken	18 (1.9)	261 (27.2)		135 (34.6)	126 (22.1)	
The number of days examinees are not allowed to undergo check-ups after a COVID-19 infection	No restrictions	N/A	N/A	N/A	48 (12.3)	45 (7.9)	<0.001
	5 days	N/A	N/A		216 (55.4)	191 (33.6)	
	7 days	N/A	N/A		59 (15.1)	140 (24.6)	
	≥10 days	N/A	N/A		64 (16.4)	188 (33.0)	
	Others	N/A	N/A		3 (0.8)	5 (0.9)	

^a^P value comparing before and after the downgrade using McNemar’s test. ^b^P value comparing facilities annexed to hospitals and those not annexed using the χ^2^ test. FY: fiscal year; N/A: not applicable.

Pulmonary function tests were suspended in most facilities during the pandemic ^(12)^, and we investigated whether the facilities restarted pulmonary function tests, because guidelines by the eight organizations related to health check-ups were updated based on recommendations for pulmonary function tests by the Japanese Respiratory Society after the COVID-19 downgrade ^(22)^. Facilities were asked when they restarted pulmonary function tests. The most frequent answer was that they were restarted in 2023 (52.8%), followed by responses that they were never interrupted (18.2%) and that they would be restarted in 2024 (11.7%) (Supplementary Figure 3).

### Factors associated with the negative impact on employees’ mental health

Of the 959 facilities, 280 (29.2%) reported that the pandemic had a negative impact on the mental health of check-up facility employees (Table 1). We investigated factors associated with this negative impact. A negative impact on the mental health of employees was associated with being annexed to hospitals, providing inpatient and outpatient care for COVID-19, experiencing a negative financial impact in 2023, increased employee resignations, decreased new recruits in fiscal year (FY) 2023, employees complaining that COVID-19-related rules were too strict, and the presence of employees having difficulty working due to long COVID (Supplementary Table 4).

Multivariate analyses revealed that the negative impact on employees’ mental health was associated with being annexed to hospitals (odds ratio [OR] 1.69, 95% confidence interval [CI] 1.18-2.41), experiencing a negative financial impact in 2023 (OR 1.97, CI 1.42-2.75), employees complaining that COVID-19-related rules were too strict (OR 2.96, CI 1.77-4.96), and the presence of employees having difficulty working due to long COVID (OR 3.39, CI 2.03-5.66) (Table 3).

**Table 3.** Multivariate Logistic Regression Model for the Negative Impact on Mental Health of Employees.

		Univariate model		Multivariate model	
		Odds ratio (95% CI)	P value	Odds ratio (95% CI)	P value
(Intercept)		-	-	0.05 (0.02-0.14)	<0.001
Type of facility	Not annexed to hospitals	Ref		Ref	
	Annexed to hospitals	1.67 (1.25-2.24)	<0.001	1.69 (1.18-2.41)	0.004
Area	Kanto	Ref		Ref	
	Hokkaido	0.78 (0.27-2.22)	0.641	0.56 (0.17-1.85)	0.344
	Tohoku	1.18 (0.65-2.17)	0.582	1.11 (0.59-2.12)	0.743
	Chubu	0.69 (0.46-1.04)	0.079	0.64 (0.41-0.99)	0.047
	Kansai	0.97 (0.66-1.43)	0.893	1.03 (0.68-1.56)	0.874
	Chugoku	0.66 (0.37-1.20)	0.172	0.60 (0.32-1.12)	0.106
	Shikoku	0.20 (0.05-0.86)	0.031	0.23 (0.05-1.02)	0.053
	Kyushu	1.01 (0.60-1.70)	0.960	1.00 (0.58-1.74)	0.995
	Okinawa	4.37 (1.07-17.8)	0.040	3.02 (0.65-13.98)	0.157
Location of facilities	Locations outside ordinance-designated cities or special wards in Tokyo	Ref		Ref	
	Ordinance-designated cities or special wards in Tokyo	1.01 (0.76-1.34)	0.960	1.03 (0.74-1.42)	0.864
Public or private institutions	Private	Ref		Ref	
	Public	1.10 (0.77-1.56)	0.604	0.93 (0.63-1.38)	0.720
Number of employees	1-10	Ref		Ref	
	11-20	1.27 (0.85-1.89)	0.250	0.45 (0.24-0.86)	0.015
	21-40	1.28 (0.86-1.89)	0.227	0.81 (0.45-1.46)	0.490
	>40	1.01 (0.66-1.54)	0.960	0.64 (0.32-1.30)	0.216
Negative financial impact in 2023	Yes	2.08 (1.53-2.83)	<0.001	1.97 (1.42-2.75)	<0.001
Employees complain that COVID-19-related rules are too strict	Yes	2.89 (1.78-4.68)	<0.001	2.96 (1.77-4.96)	<0.001
Some employees have difficulty working due to long COVID	Yes	3.57 (2.20-5.79)	<0.001	3.39 (2.03-5.66)	<0.001
Wearing masks indoors during personal time	Personal choice	Ref		Ref	
	Compulsory or recommended	1.36 (1.00-1.84)	0.048	1.23 (0.86-1.76)	0.250
Taking COVID-19 tests for fever	Personal choice	Ref		Ref	
	Compulsory or recommended	0.93 (0.64-1.36)	0.709	0.83 (0.54-1.27)	0.384
When family members are infected with COVID-19	No restriction/allowed to work if they are not sick	Ref		Ref	
	Employees are not allowed to work for a certain period	1.05 (0.75-1.47)	0.772	1.01 (0.70-1.46)	0.963
Employees are recommended to receive the COVID-19 vaccination	Yes	1.03 (0.78-1.36)	0.833	0.84 (0.61-1.14)	0.255
Organizational social gathering among employees involving meals	Not restricted	Ref		Ref	
	Restricted/Not restricted but rarely held	1.19 (0.88-1.60)	0.263	1.28 (0.87-1.90)	0.215
Private meals with people other than colleagues	Not restricted	Ref		Ref	
	Restricted/Not restricted but rarely held	1.04 (0.77-1.41)	0.802	0.81 (0.56-1.17)	0.267
Organizational social gathering among employees involving meals held in FY2019/FY2023	FY2019: no FY2023: no	Ref		Ref	
	FY2019: no FY2023: yes	0.83 (0.55-1.24)	0.360	0.95 (0.61-1.51)	0.841
	FY2019: yes FY2023: no	0.88 (0.60-1.29)	0.505	0.89 (0.59-1.34)	0.571
	FY2019: yes FY2023: yes	0.86 (0.58-1.28)	0.457	0.92 (0.58-1.45)	0.715

FY: fiscal year; Ref: reference; CI: confidence interval.

### Association between employee attrition and the negative impact on employees’ mental health

**Figure 3.**
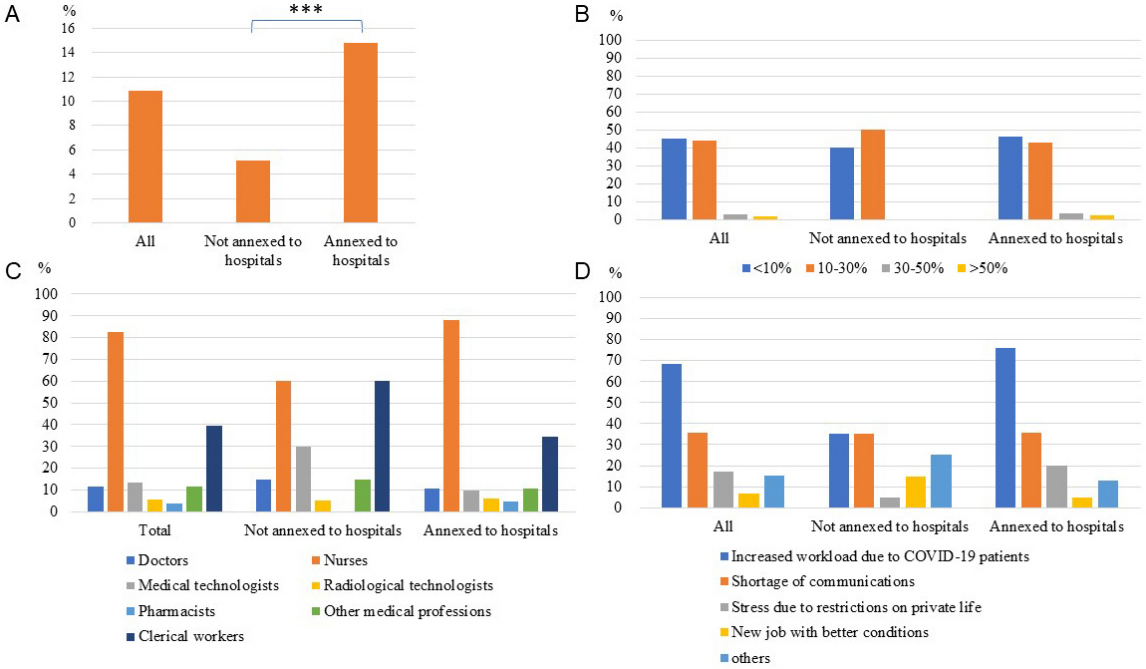
A: Proportion of facilities reporting increased employee resignations compared to the pre-pandemic period among all participating facilities (n = 959), those annexed to hospitals (n = 569), and those not annexed to hospitals (n = 390). *** P < 0.001 B: Extent of increase in resignations compared to the pre-pandemic period, C: the job types with increased resignations, and D: the main reasons for the increase among all facilities reporting increased employee resignations (n = 104), those annexed to hospitals (n = 84) and those not annexed to hospitals (n = 20).

Next, we assessed the impact of the pandemic on employee attrition and examined whether attrition was associated with the negative impact on employees’ mental health. Increased employee resignations compared to the pre-pandemic period were reported by 104 facilities (10.8%); while 5.1% of facilities not annexed to hospitals reported an increase, 14.8% of those annexed to hospitals did. Of the 104 facilities, 47 (45.2%), 46 (44.2%), 3 (2.9%) and 2 (1.9%) reported that resignations increased by <10%, 10%-30%, 30%-50%, and >50%, respectively (Figure 3 and Table 4). Among the job types with increased resignations, the highest frequency was observed among nurses (82.7%), followed by clerical workers (39.4%), clinical laboratory technologists (13.5%), doctors (11.5%), and other medical professionals (11.5%). The main reasons for this increase were increased workload due to patients with COVID-19 (68.3%), shortage of communications (35.6%), stress due to restrictions in private life (17.3%), and new jobs with better conditions (6.7%). Other reasons included an increased overall workload, a deterioration of working conditions due to the worsening financial situation, and a gap between actual work and expectations caused by a lack of pre-employment training.

**Table 4.** Rate of Increase, Job Types, and the Reasons for Increased Employee Resignations.

		Total (n = 104)	Not annexed to hospitals (n = 20)	Annexed to hospitals (n = 84)	P value
Rate of increase in resignations compared to the pre-pandemic period (single answer)	<10%	47 (45.2)	8 (40.0)	39 (46.4)	0.675
	10%-30%	46 (44.2)	10 (50.0)	36 (42.9)	
	30%-50%	3 (2.9)	0 (0.0)	3 (3.6)	
	>50%	2 (1.9)	0 (0.0)	2 (2.4)	
Job types with increased resignations (multiple answers)	Doctors	12 (11.5)	3 (15.0)	9 (10.7)	0.881
	Nurses	86 (82.7)	12 (60.0)	74 (88.1)	0.008
	Clinical laboratory technologists	14 (13.5)	6 (30.0)	8 (9.5)	0.041
	Radiologic technologists	6 (5.8)	1 (5.0)	5 (6.0)	>0.999
	Pharmacists	4 (3.8)	0 (0.0)	4 (4.8)	0.728
	Other medical professionals	12 (11.5)	3 (15.0)	9 (10.7)	0.881
	Clerical workers	41 (39.4)	12 (60.0)	29 (34.5)	0.066
Reasons for increased resignation (multiple answers)	Increased workload due to patients with COVID-19	71 (68.3)	7 (35.0)	64 (76.2)	0.001
	Shortage of communications	37 (35.6)	7 (35.0)	30 (35.7)	>0.999
	Stress due to restrictions in private life	18 (17.3)	1 (5.0)	17 (20.2)	0.197
	New job with better conditions	7 (6.7)	3 (15.0)	4 (4.8)	0.252
	Other reasons (below)	16 (15.4)	5 (25.0)	11 (13.1)	0.326
Other reasons (open-ended response)	Increased overall workload	2 (1.9)			
	Deterioration of working conditions due to the worsening financial situation	2 (1.9)			
	A gap between the actual work and expectations due to the lack of pre-employment training	2 (1.9)			

FY: fiscal year.

We evaluated the factors associated with an increase in employee resignations. An increase in employee resignations was associated with a negative impact on employees’ mental health, in addition to being annexed to hospitals, providing inpatient and outpatient care for patients with COVID-19, a negative financial impact in 2023, and a decrease in new recruits (Supplementary Table 5). Holding organizational social gatherings with meals (e.g., hospital-wide year-end parties, department-wide farewell parties) in FY2019 but not in FY2023 was associated with an increase in employee resignations. Conversely, not holding such gatherings in FY2019 but doing so in FY2023 was negatively associated with an increase in employee resignations.

An increase in employee resignations was strongly associated with a negative impact on the mental health of employees, even after multivariable adjustment (Table 5). Compared to not holding organizational social gatherings in either FY2019 or FY2023, holding one in FY2019 but not in FY2023 was marginally associated with an increase in employee resignations (P = 0.0503), while not holding one in FY2019 but in FY2023 was negatively associated.

**Table 5.** Multivariate Logistic Regression Model for Increased Resignation of Employees.

		Univariate model		Multivariate model	
		Odds ratio (95% CI)	P value	Odds ratio (95% CI)	P value
(Intercept)		-	-	0.05 (0.02-0.14)	<0.001
Type of facility	Not annexed to hospitals	Ref		Ref	
	Annexed to hospitals	3.20 (1.93-5.31)	<0.001	2.83 (1.56-5.13)	<0.001
Area	Kanto	Ref		Ref	
	Hokkaido	3.02 (1.03-8.89)	0.045	2.85 (0.85-9.54)	0.090
	Tohoku	1.06 (0.42-2.65)	0.906	0.91 (0.34-2.43)	0.852
	Chubu	1.12 (0.63-2.00)	0.690	0.99 (0.53-1.86)	0.977
	Kansai	0.84 (0.46-1.55)	0.577	0.84 (0.44-1.62)	0.611
	Chugoku	0.90 (0.38-2.11)	0.803	0.92 (0.37-2.31)	0.862
	Shikoku	0.37 (0.05-2.81)	0.335	0.51 (0.06-4.12)	0.529
	Kyushu	1.04 (0.48-2.27)	0.916	0.95 (0.41-2.20)	0.913
	Okinawa	4.23 (1.01-17.66)	0.048	2.39 (0.42-13.44)	0.324
Location of facilities	Locations outside ordinance-designated cities or special wards in Tokyo	Ref		Ref	
	Ordinance-designated cities or special wards in Tokyo vs others	0.95 (0.63-1.45)	0.815	1.25 (0.77-2.02)	0.376
Public or private institutions	Private	Ref		Ref	
	Public	1.58 (0.99-2.54)	0.056	1.13 (0.66-1.92)	0.660
Number of employees	1-10	Ref		Ref	
	11-20	0.60 (0.33-1.09)	0.095	0.45 (0.24-0.86)	0.015
	21-40	1.01 (0.60-1.70)	0.976	0.81 (0.45-1.46)	0.490
	>40	0.58 (0.31-1.08)	0.086	0.64 (0.32-1.30)	0.216
Negative financial impact in 2023	Yes	1.75 (1.13-2.69)	0.012	1.35 (0.84-2.18)	0.213
Employees complain that COVID-19 related rules are too strict	Yes	1.89 (1.00-3.58)	0.0498	1.20 (0.59-2.45)	0.615
Some employees have difficulty in working due to long COVID	Yes	1.31 (0.65-2.65)	0.443	0.79 (0.35-1.75)	0.558
Wearing masks indoors in private time	Personal choice	Ref		Ref	
	Compulsory or recommended	1.03 (0.67-1.59)	0.896	0.76 (0.45-1.26)	0.285
Taking COVID-19 tests for fever	Personal choice	Ref		Ref	
	Compulsory or recommended	0.95 (0.55-1.65)	0.859	0.87 (0.46-1.64)	0.660
When family members are infected with COVID-19	No restriction/allowed to work if they are not sick	Ref		Ref	
	Employees are not allowed to work for a certain period	0.95 (0.58-1.57)	0.846	0.83 (0.48-1.44)	0.505
Employees are recommended to receive the COVID-19 vaccination	Yes	1.54 (1.02-2.33)	0.040	1.41 (0.88-2.24)	0.150
Organizational social gathering among employees involving meals	Not restricted	Ref		Ref	
	Restricted or rarely held	1.31 (0.84-2.05)	0.232	1.01 (0.55-1.85)	0.972
Private meals with people other than colleagues	Not restricted	Ref		Ref	
	Restricted or rarely held	0.98 (0.63-1.53)	0.938	0.94 (0.55-1.60)	0.814
Organizational social gathering among employees involving meals held in FY2019/FY2023	FY2019: no FY2023: no	Ref		Ref	
	FY2019: no FY2023: yes	0.38 (0.17-0.84)	0.017	0.35 (0.15-0.83)	0.017
	FY2019: yes FY2023: no	1.81 (1.05-3.12)	0.032	1.79 (1.00-3.21)	0.0503
	FY2019: yes FY2023: yes	1.24 (0.69-2.24)	0.472	1.14 (0.58-2.25)	0.703
Negative impact on the mental health of employees	Yes	3.44 (2.27-5.21)	<0.001	3.26 (2.07-5.15)	<0.001

FY: fiscal year; Ref: reference; CI: confidence interval.

### Negative financial impact on the management in 2023

We previously reported that approximately 60% of the facilities reported a negative financial impact on the management of health check-ups, even in 2022 ^(8)^. Therefore, we assessed the extent of this impact in 2023. A negative financial impact on the management of check-up facilities in 2023 was reported by 235 (24.5%) facilities, with no significant difference between hospital-annexed and non-annexed facilities (Table 1). The most frequent reason for the negative financial impact was that examinees who avoided visits during the COVID-19 pandemic had not returned (60.4%), followed by increased costs due to precautions against COVID-19 (51.5%), limiting appointments due to staff shortages (24.7%) and other reasons (17.0%), including last-minute cancellations due to sickness (5.5%) and limiting appointments as a precaution against COVID-19 (4.3%) (Supplementary Table 6).

## Discussion

In this nationwide follow-up survey of healthcare facilities, conducted after the COVID-19 downgrade in 2023, we report that the number of examinees undergoing non-mandatory cancer screenings provided by local governments finally recovered to the pre-pandemic level in 2023 (Figure 1). However, gastric cancer screenings remained below pre-pandemic levels, raising concerns about delayed diagnoses. Additionally, nearly 30% of the facilities reported a negative impact on employees’ mental health, with factors such as being annexed to hospitals and employees’ complaints about strict COVID-19-related rules associated with this negative impact. Notably, increased employee resignations were also linked to this negative mental health impact.

### Impact of the COVID-19 pandemic and downgrade on the number of examinees undergoing check-ups

Cancer screening programs were disrupted during the early phase of the pandemic globally ^(23), (24), (25)^. More recent studies revealed that cancer screenings had not fully recovered to the pre-pandemic levels even in 2022 ^(7), (8), (26)^. In our previous surveys, we found that while the number of examinees undergoing mandatory check-ups recovered by 2021 from the decline during the COVID-19 pandemic, the number of those undergoing non-mandatory check-ups remained low in 2021 ^(12)^, a tendency that persisted into 2022 ^(8)^. The result that it has taken three years for these screenings to recover to the pre-pandemic levels, raises concerns about delays in diagnosis and highlights a challenge for future pandemics. It remains to be determined which characteristics of the examinees were most impacted by the pandemic. Healthcare policies should specifically target these individuals during future pandemics to improve screening rates. Targeted outreach programs emphasizing the importance of timely screenings could help improve participation rates and minimize delays in diagnosis.

### Factors associated with the negative impact of the pandemic on employees’ mental health

A negative impact on employees’ mental health due to the COVID-19 pandemic was reported by 29.2% of the facilities; 23.1% of those not annexed to hospitals and 33.4% of those annexed to hospitals reported this negative impact. The pandemic imposed unprecedented stress on healthcare professionals, and a wide range of mental health problems among healthcare workers, such as depression, anxiety, post-traumatic stress, and insomnia, have been reported ^(16), (17)^. Reported risk factors included being female, working in high-risk units, and providing direct care for patients with COVID-19 ^(16)^, though non-frontline healthcare workers were also experiencing psychological distress ^(27)^. Although our study focused on health check-up facilities, where employees are generally not in high-risk settings, our findings suggest that many of these staff members were nevertheless impacted by the hospital pandemic environment. Approximately 60% of the participating facilities were annexed to hospitals, and of these, over 80% provided both inpatient and outpatient care for patients with COVID-19. Importantly, nearly 80% of annexed facilities reported personnel transfers between the check-up facility and the hospital. Furthermore, our previous survey indicated that approximately half of the annexed facilities required health check-up staff to assist with COVID-19-related care ^(8)^. These results suggest that, during the pandemic, personnel working in health check-up facilities were often involved in clinical duties or subjected to hospital-level infection control measures, which may have contributed to their mental health burden. The negative impact on employees’ mental health was associated with being annexed to hospitals, experiencing a negative financial impact in 2023, employees complaining that COVID-19-related rules were too strict, and the presence of employees having difficulty working due to long COVID (Table 3). Although we did not find significant associations with specific COVID-19-related rules, such as wearing masks, taking COVID-19 tests, or holding meals and gatherings, previous reports have suggested that COVID-19-related restrictions were associated with mental health problems ^(19)^. As healthcare workers were subject to stricter restrictions for a longer period than others, it is possible that these restrictions contributed to the negative impact on their mental health. Indeed, our results revealed that employees in facilities annexed to hospitals were subject to stricter COVID-19-related restrictions in almost every aspect compared to those in facilities not annexed to hospitals, even after the downgrade. Long COVID has also been reported to affect mental health ^(28)^.

### The association between the pandemic’s negative impact on employees’ mental health and attrition

Understanding the factors influencing attrition and taking measures to prevent it is crucial, especially during healthcare crises such as pandemics, to ensure the continuity of healthcare services. Mental health is a key factor, but other factors such as increased workload, financial pressures, and workplace relationships also contribute to attrition. A recent study analyzing factors associated with the attrition of UK healthcare workers since the COVID-19 pandemic reported a strong association between the desire to leave healthcare and mental health measures ^(15)^. This highlights the need to integrate mental health support to mitigate attrition. Adverse mental health can lead to increased attrition among healthcare workers ^(17)^, and reports have suggested that the intention to leave one’s job among healthcare professionals was high during the pandemic ^(29), (30), (31), (32)^. Consistent with previous reports, the negative impact on employees’ mental health was strongly associated with increased resignations in this study. In addition, holding organizational social gatherings involving meals in FY2019 but not in FY2023 was marginally associated with increased resignations, while not holding such gatherings in FY2019 but doing so in FY2023 was negatively associated with increased resignations. This suggests that more active communication among co-workers might have helped prevent employee resignations. The highest frequency of increased resignations was observed among nurses, and the most common reason for increased resignations was the increased workload due to patients with COVID-19. Providing mental health support and fostering active communication among employees may be crucial for preventing attrition during future pandemics.

### Strengths and limitations

The key strength of this study is that it was a large-scale nationwide survey conducted in Japan, achieving a response rate of over 60%. We uncovered the impact of the COVID-19 pandemic on health check-ups up to 2023, covering the periods before and after the COVID-19 downgrade. Moreover, we compared the precautions against COVID-19 and COVID-19-related rules during the year before and the period after the COVID-19 downgrade. To the best of our knowledge, this is the first large-scale study to investigate the negative impact on the mental health of check-up facility employees due to the COVID-19 pandemic.

This study had several limitations. First, the survey was completed by a representative from each facility; therefore, the negative impact on employees’ mental health may have been influenced by their personal impressions. Second, due to the unique nature of Japan’s health check-up system, these results may not be applicable to other regions. Third, there may be selection bias in the participating facilities, as the exact total number of facilities providing health check-ups, including non-members of Japan Society of Ningen Dock and Preventive Medical Care, is unavailable.

### Conclusions

Although the number of examinees participating in non-mandatory cancer screenings provided by local governments returned to pre-pandemic levels in 2023, the number for gastric cancer screenings remained low. An increase in employee resignations in check-up facilities was associated with a negative impact on the mental health of employees, which in turn was linked to being annexed to hospitals, experiencing a negative financial impact in 2023, employees complaining that COVID-19-related rules were too strict, and the presence of employees having difficulty working due to long COVID. Providing mental health support to healthcare workers, including those engaged in preventive medicine, is especially important during pandemics to prevent attrition and maintain healthcare services.

## Article Information

### Conflicts of Interest

Satoko Yamaguchi, Akira Okada, Reiko Inoue, and Takashi Kadowaki are members of the Department of Prevention of Diabetes and Lifestyle-related Diseases, which is a cooperative program between the University of Tokyo and Asahi Mutual Life Insurance Company. Tomofumi Atarashi, Shigeru Nasu, Toshimasa Yamauchi, Yasuji Arase, Takao Aizawa, and Masaomi Nangaku declare no competing interests.

### Sources of Funding

This work was supported by MHLW Research on Emerging and Re-emerging Infectious Diseases and Immunization (Program Grant Numbers JPMH23HA2011 and JPMH24HA2015). The funding organization has no role in the design of the study, analysis, interpretation of data, or writing the manuscript.

### Acknowledgement

We would like to thank all the participating facilities for taking the time to respond to the questionnaire. We thank Ms. Emi Yoshikawa and Mr. Shoji Negishi of Japan Society of Ningen Dock and Preventive Medical Care for their assistance in this study.

### Author Contributions

Satoko Yamaguchi, Tomofumi Atarashi, Akira Okada, Shigeru Nasu, Masaomi Nangaku, and Takashi Kadowaki designed the study. Tomofumi Atarashi and Shigeru Nasu acquired data. Satoko Yamaguchi analyzed the data. Satoko Yamaguchi and Takashi Kadowaki wrote the first draft of the manuscript. All authors contributed to the interpretation of data and reviewed, revised, and approved the final manuscript. Satoko Yamaguchi and Tomofumi Atarashi contributed equally as first authors.

### Approval by Institutional Review Board (IRB)

This study was approved by the institutional review board of the Graduate School of Medicine at the University of Tokyo (2018030NI) and was performed in accordance with the relevant guidelines and regulations (the Declaration of Helsinki). The requirement for informed consent was waived because the facility-based survey did not contain any personal information.

## Supplement

Supplementary Materials
