# Supplementary material for: Impact of the COVID-19 Pandemic on Health Check-ups before and after the COVID-19 Downgrade and Employees’ Mental Health at Health Check-up Facilities: The Third Nationwide Survey of Healthcare Facilities in Japan Society of Ningen Dock and Preventive Medical Care
Source: JMA J. 2025 Jun 27;8(3):911–24. doi: 10.31662/jmaj.2024-0337 (PMC12328909; doi:10.31662/jmaj.2024-0337)
Supplement: Supplementary Materials [file 2433-3298-8-3-0911-s001.pdf]

Impact of the COVID-19 Pandemic on Health Check-ups before and after the COVID-19 Downgrade and Employees' Mental Health at Health Check-up Facilities: The Third Nationwide Survey of Healthcare Facilities in Japan Society of Ningen Dock and Preventive Medical Care

Supplementary materials

Supplementary Table 1. Characteristics of the member facilities of Japan Society of Ningen Dock and Preventive Medical Care by participation in the current study

		Total (n = 1813)	Participation in this study		P value[a]
			No (n = 833)	Yes (n = 980)	
Area[b]	Kanto	631 (34.8)	294 (35.3)	337 (34.4)	0.008
	Hokkaido	55 (3.0)	35 (4.2)	20 (2.0)	
	Tohoku	96 (5.3)	41 (4.9)	55 (5.6)	
	Chubu	307 (16.9)	125 (15.0)	182 (18.6)	
	Kansai	354 (19.5)	160 (19.2)	194 (19.8)	
	Chugoku	117 (6.5)	43 (5.2)	74 (7.6)	
	Shikoku	54 (3.0)	30 (3.6)	24 (2.4)	
	Kyushu	174 (9.6)	91 (10.9)	83 (8.5)	
	Okinawa	25 (1.4)	14 (1.7)	11 (1.1)	
Location of facilities	Ordinance-designated cities/special wards in Tokyo	700 (38.6)	314 (37.7)	386 (39.4)	0.491
Public or private institutions	Public	359 (19.8)	171 (20.5)	188 (19.2)	0.511

[a]P-value comparing the facilities that participated and those that did not participate in this study using the chi-square test
[b]Areas of Japan: Japan comprises the four main islands of Hokkaido, Honshu, Shikoku, and Kyushu. Honshu, the largest island, is divided into Kanto (which includes the Greater Tokyo area), Tohoku, Chubu, Kansai and Chugoku regions. Okinawa is the southernmost prefecture that includes the fifth largest island.

Supplementary Table 2. Annual number of check-ups between 2019 and 2023 in 430 facilities

	2019 (% change)	2020 (% change)	2021 (% change)	2022 (% change)	2023 (% change)
All check-ups	16,627,394 (0.0)	14798762 (-11.0)	16,265,637 (-2.2)	16,396,439 (-1.4)	16,459,337 (-1.0)
Check-ups based on Industrial Safety and Health Act	2,008,831 (0.0)	1,906,130 (-5.1)	2,086,095 (3.8)	2,128,990 (6.0)	2,170,445 (8.0)
Check-ups for prevention of lifestyle-related diseases	1,973,432 (0.0)	1,877,944 (-4.8)	2,107,909 (6.8)	2,127,354 (7.8)	2,173,177 (10.1)
Comprehensive check-ups (Ningen Dock)[a]	2,213,346 (0.0)	1,927,971 (-12.9)	2,147,118 (-3.0)	2,116,636 (-4.4)	2,187,222 (-1.2)
Specific Health Check-ups alone[b]	363,311 (0.0)	299,646 (-17.5)	344,633 (-5.1)	353,834 (-2.6)	349,258 (-3.9)
Cancer screenings by local governments	1,523,643 (0.0)	1,319,434 (-13.4)	1,428,960 (-6.2)	1,442,165 (-5.3)	1,537,958 (0.9)
Other in-facility check-ups[c]	853,973 (0.0)	775,576 (-9.2)	842,705 (-1.3)	825,053 (-3.4)	792,013 (-7.3)
Check-ups using mobile medical vehicles	7,690,858 (0.0)	6,692,061 (-13.0)	7,308,217 (-5.0)	7,402,407 (-3.8)	7,249,264 (-5.7)

[a]Comprehensive check-ups (Ningen Dock) as defined by Japan Society of Ningen Dock and Preventive Medical Care

[b]"Specific Health Check-ups alone" refers to Specific Health Check-ups without mandatory check-ups. All items in Specific Health Check-ups are included in the "Check-ups based on Industrial Safety and Health Act," "Check-ups for prevention of lifestyle-related diseases," and "Comprehensive check-ups Ningen Dock."

[c]Includes comprehensive check-ups (Ningen Dock) that do not fulfil the definition of Japan Society of Ningen Dock and Preventive Medical Care

Supplementary Table 3. Precautions against COVID-19 to perform in-facility check-ups before and after the COVID-19 downgrade

	Before vs after COVID-19 downgrade			After COVID-19 downgrade by facility type		
	Before COVID-19 downgrade (n=959)	After COVID-19 downgrade (n=959)	P value[a]	Not annexed to hospitals (n=390)	Annexed to hospitals (n=569)	P value[b]
Sufficient ventilation	939 (97.9)	870 (90.7)	<0.001	356 (91.3)	514 (90.3)	0.701
Handwashing/hand disinfection	959 (100.0)	899 (93.7)	<0.001	353 (90.5)	546 (96.0)	0.001
Disinfection of high-touch surfaces	950 (99.1)	826 (86.1)	<0.001	320 (82.1)	506 (88.9)	0.003
Installing partitions	831 (86.7)	406 (42.3)	<0.001	174 (44.6)	232 (40.8)	0.264
Checking the body temperature of the employees	916 (95.5)	495 (51.6)	<0.001	174 (44.6)	321 (56.4)	<0.001
Checking the body temperature of the examinees	942 (98.2)	686 (71.5)	<0.001	246 (63.1)	440 (77.3)	<0.001
Asking examinees about recent COVID-19 infection	873 (91.0)	563 (58.7)	<0.001	172 (44.1)	391 (68.7)	<0.001
Asking examinees about health conditions (fever, cold symptoms)	926 (96.6)	775 (80.8)	<0.001	277 (71.0)	498 (87.5)	<0.001
Employees use either face shields, eye shields, or goggles	688 (71.7)	275 (28.7)	<0.001	97 (24.9)	178 (31.3)	0.037
Employees are recommended to get vaccinated against COVID-19	893 (93.1)	471 (49.1)	<0.001	151 (38.7)	320 (56.2)	<0.001
None of the above	0 (0.0)	20 (2.1)	<0.001	7 (1.8)	13 (2.3)	0.771

[a]P value comparing before and after the COVID-19 downgrade using McNemar's test.
[b]P value comparing facilities annexed to hospitals and those not annexed using the chi-square test.

Supplementary Table 4. Characteristics of facilities by negative impact on employees'mental health

		Total (n=959)	No negative impact on mental health (n=679)	Negative impact on mental health (n=280)	P value
Area[a]	Kanto	331 (34.5)	227 (33.4)	104 (37.1)	0.030
	Hokkaido	19 (2.0)	14 (2.1)	5 (1.8)	
	Tohoku	54 (5.6)	35 (5.2)	19 (6.8)	
	Chubu	179 (18.7)	136 (20.0)	43 (15.4)	
	Kansai	188 (19.6)	130 (19.1)	58 (20.7)	
	Chugoku	73 (7.6)	56 (8.2)	17 (6.1)	
	Shikoku	24 (2.5)	22 (3.2)	2 (0.7)	
	Kyushu	82 (8.6)	56 (8.2)	26 (9.3)	
	Okinawa	9 (0.9)	3 (0.4)	6 (2.1)	
Location of facilities	Ordinance-designated cities/special wards in Tokyo	379 (39.5)	268 (39.5)	111 (39.6)	>0.999
Public or private institutions	Public	182 (19.0)	126 (18.6)	56 (20.0)	0.669
Type of facilities	Not annexed to hospitals	390 (40.7)	300 (44.2)	90 (32.1)	<0.001
	Annexed to hospitals	569 (59.3)	379 (55.8)	190 (67.9)	
Number of employees	1–10	219 (22.8)	161 (23.7)	58 (20.7)	0.444
	11–20	249 (26.0)	171 (25.2)	78 (27.9)	
	21–40	270 (28.2)	185 (27.2)	85 (30.4)	
	>40	221 (23.0)	162 (23.9)	59 (21.1)	
Providing inpatient care for patients with COVID-19	Yes	482 (50.3)	324 (47.7)	158 (56.4)	0.017
Providing outpatient care for patients with COVID-19	Yes	648 (67.6)	443 (65.2)	205 (73.2)	0.020
Negative financial impact in 2023	Yes	235 (24.5)	138 (20.3)	97 (34.6)	<0.001
Resignation of employees compared to pre-pandemic time	Increased	104 (10.8)	47 (6.9)	57 (20.4)	<0.001
Number of new recruits in 2023 compared to 2019	Decreased	123 (12.8)	75 (11.0)	48 (17.1)	0.014
Employees complain that COVID-19 related rules are too strict	Yes	73 (7.6)	35 (5.2)	38 (13.6)	<0.001
Some employees have difficulty working due to long COVID	Yes	74 (7.7)	32 (4.7)	42 (15.0)	<0.001
Wearing masks at work	Compulsory or recommended	955 (99.6)	675 (99.4)	280 (100)	0.462
Wearing masks during commuting	Compulsory or recommended	741 (77.3)	519 (76.4)	222 (79.3)	0.383
Wearing masks indoors during personal time	Compulsory or recommended	640 (66.7)	440 (64.8)	200 (71.4)	0.057
Taking COVID-19 tests for fever	Compulsory or recommended	808 (84.3)	574 (84.5)	234 (83.6)	0.783
When family members are infected with COVID-19	Employees are not allowed to work for a certain period	210 (21.9)	147 (21.6)	63 (22.5)	0.839
Vaccination against COVID-19	Recommended	471 (49.1)	332 (48.9)	139 (49.6)	0.889
Organizational social gathering among employees involving meals (hospital-wide year-end party, department-wide farewell party)	Restricted or rarely held	632 (65.9)	440 (64.8)	192 (68.6)	0.296
Private meals and gatherings with colleagues	Restricted or rarely held	452 (47.1)	318 (46.8)	134 (47.9)	0.828
Private meals with people other than	Restricted or rarely held	289 (30.1)	203 (29.9)	86 (30.7)	0.862

colleagues					
Organizational social gathering among employees involving meals held in FY2019/ FY2023	FY2019:no FY2023:no	224 (23.4)	153 (22.5)	71 (25.4)	0.809
	FY2019:no FY2023:yes	227 (23.7)	164 (24.2)	63 (22.5)	
	FY2019:yes FY2023:no	273 (28.5)	194 (28.6)	79 (28.2)	
	FY2019:yes FY2023:yes	235 (24.5)	168 (24.7)	67 (23.9)	

FY: fiscal year

Supplementary Table 5. Characteristics of facilities based on increased employee resignations

		Total (n = 959)	No increase in resignations (n = 855)	Increase in resignations (n = 104)	*P* value
Area [a]	Kanto	331 (34.5)	296 (34.6)	35 (33.7)	0.178
	Hokkaido	19 (2)	14 (1.6)	5 (4.8)	
	Tohoku	54 (5.6)	48 (5.6)	6 (5.8)	
	Chubu	179 (18.7)	158 (18.5)	21 (20.2)	
	Kansai	188 (19.6)	171 (20)	17 (16.3)	
	Chugoku	73 (7.6)	66 (7.7)	7 (6.7)	
	Shikoku	24 (2.5)	23 (2.7)	1 (1.0)	
	Kyushu	82 (8.6)	73 (8.5)	9 (8.7)	
	Okinawa	9 (0.9)	6 (0.7)	3 (2.9)	
Location of facilities	Ordinance-designated cities/special wards in Tokyo	379 (39.5)	339 (39.6)	40 (38.5)	0.898
Public or private institutions	Public	182 (19.0)	155 (18.1)	27 (26.0)	0.073
Type of facilities	Not annexed to hospitals	390 (40.7)	370 (43.3)	20 (19.2)	<0.001
	Annexed to hospitals	569 (59.3)	485 (56.7)	84 (80.8)	
Number of employees	1–10	219 (22.8)	190 (22.2)	29 (27.9)	0.103
	11–20	249 (26.0)	228 (26.7)	21 (20.2)	
	21–40	270 (28.2)	234 (27.4)	36 (34.6)	
	>40	221 (23.0)	203 (23.7)	18 (17.3)	
Providing inpatient care for patients with COVID-19	Yes	482 (50.3)	408 (47.7)	74 (71.2)	<0.001
Providing outpatient care for patients with COVID-19	Yes	648 (67.6)	563 (65.8)	85 (81.7)	0.002
Negative financial impact in 2023	Yes	235 (24.5)	199 (23.3)	36 (34.6)	0.016
Number of new recruits in 2023 compared to 2019	Decreased	123 (12.8)	88 (10.3)	35 (33.7)	<0.001
Employees complain that COVID-19 related rules are too strict	Yes	73 (7.6)	60 (7.0)	13 (12.5)	0.073
Negative impact on mental health of check-up facility employees	Yes	280 (29.2)	223 (26.1)	57 (54.8)	<0.001
Some employees have difficulty in working due to long COVID	Yes	74 (7.7)	64 (7.5)	10 (9.6)	0.566
Wearing masks at work	Compulsory or recommended	955 (99.6)	851 (99.5)	104 (100.0)	>0.999
Wearing masks during commuting	Compulsory or recommended	741 (77.3)	660 (77.2)	81 (77.9)	0.972
Wearing masks indoors during personal time	Compulsory or recommended	640 (66.7)	570 (66.7)	70 (67.3)	0.983
Taking COVID-19 tests for fever	Compulsory or recommended	808 (84.3)	721 (84.3)	87 (83.7)	0.972
When family members are infected with COVID-19	Employees are not allowed to work for a certain period	210 (21.9)	188 (22.0)	22 (21.2)	0.945
Vaccination against COVID-19	Recommended	471 (49.1)	410 (48.0)	61 (58.7)	0.0503
Organizational social gathering among employees involving meals (hospital-wide year-end party, department-wide farewell party)	Restricted or rarely held	632 (65.9)	558 (65.3)	74 (71.2)	0.277
Private meals and gatherings with colleagues	Restricted or rarely held	452 (47.1)	398 (46.5)	54 (51.9)	0.351
Private meals with people other than colleagues	Restricted or rarely held	289 (30.1)	258 (30.2)	31 (29.8)	>0.999
Organizational social gathering	FY2019: no FY2023: no	224 (23.4)	202 (23.6)	22 (21.2)	<0.001

among employees involving meals held in FY2019/FY2023	FY2019: no FY2023: yes	227 (23.7)	218 (25.5)	9 (8.7)	
	FY2019: yes FY2023: no	273 (28.5)	228 (26.7)	45 (43.3)	
	FY2019: yes FY2023: yes	235 (24.5)	207 (24.2)	28 (26.9)	

FY: fiscal year

Supplementary Table 6. Reasons for the negative financial impact on the management of check-up facilities in 2023

	Total (n = 235)	Facilities not annexed to hospitals (n = 83)	Facilities annexed to hospitals (n = 152)	P value
Examinees who avoided visits during the COVID-19 pandemic have not returned	142 (60.4)	56 (67.5)	86 (56.6)	0.136
Limiting appointments due to staff shortages	58 (24.7)	13 (15.7)	45 (29.6)	0.027
Increased cost due to precautions against COVID-19	121 (51.5)	58 (69.9)	63 (41.4)	<0.001
Other reasons	40 (17.0)	15 (18.1)	25 (16.4)	0.892
Other reasons in detail (open-ended answers)				
Last-minute cancellation due to sickness	13 (5.5)			
Limiting appointments as a precaution against COVID-19	10 (4.3)			
The promotion of telework has led to an increase in the number of people seeking care from other healthcare facilities.	6 (2.6)			
Suspension of pulmonary function tests or endoscopies	3 (1.3)			
Increased utility costs and material expenses	2 (0.9)			
Downsizing of client companies	2 (0.9)			
Increased burden due to dealing with examinees who refuse to wear masks	2 (0.9)			
Cancellation of group health check-ups	2 (0.9)			

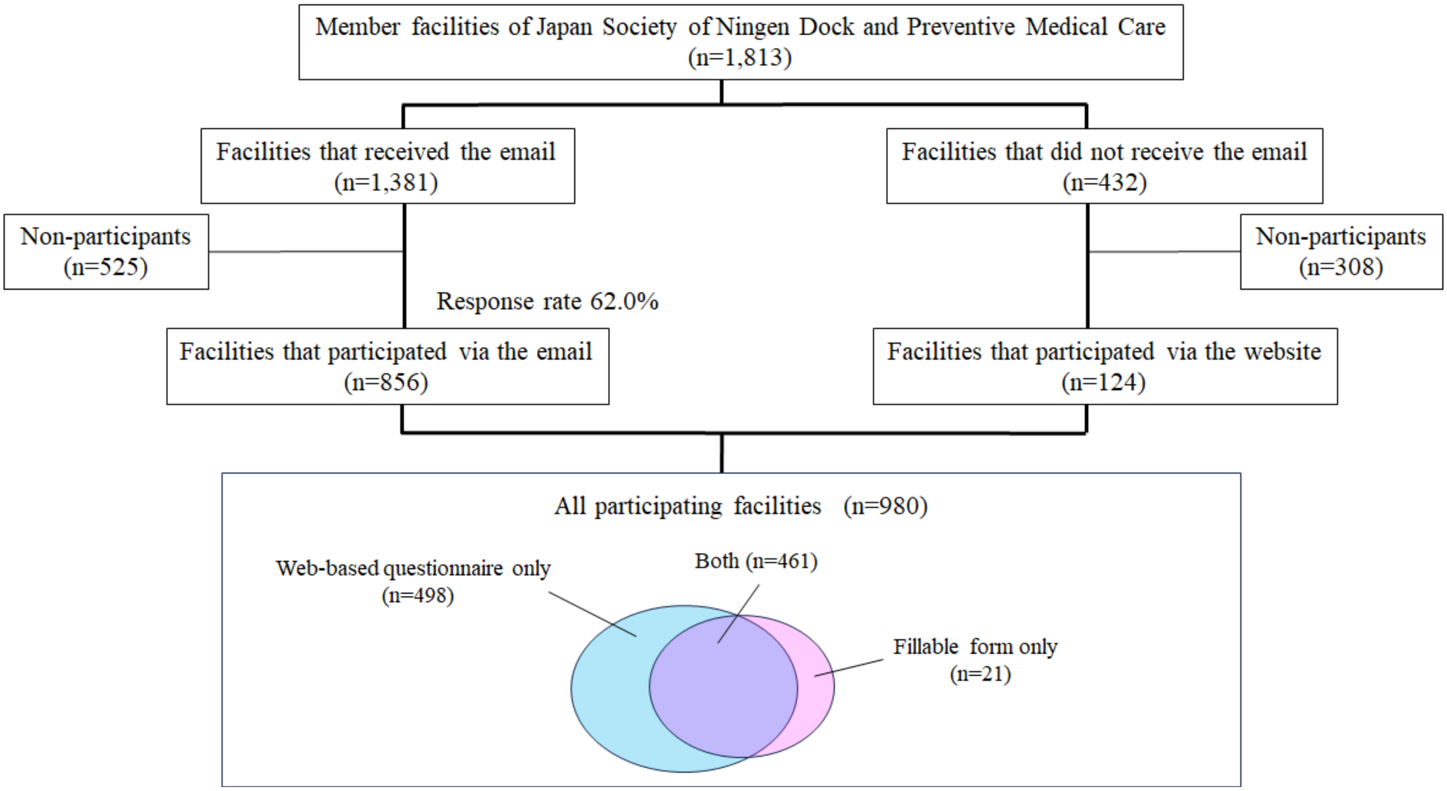

Supplementary Figure 1. Flowchart of the study.

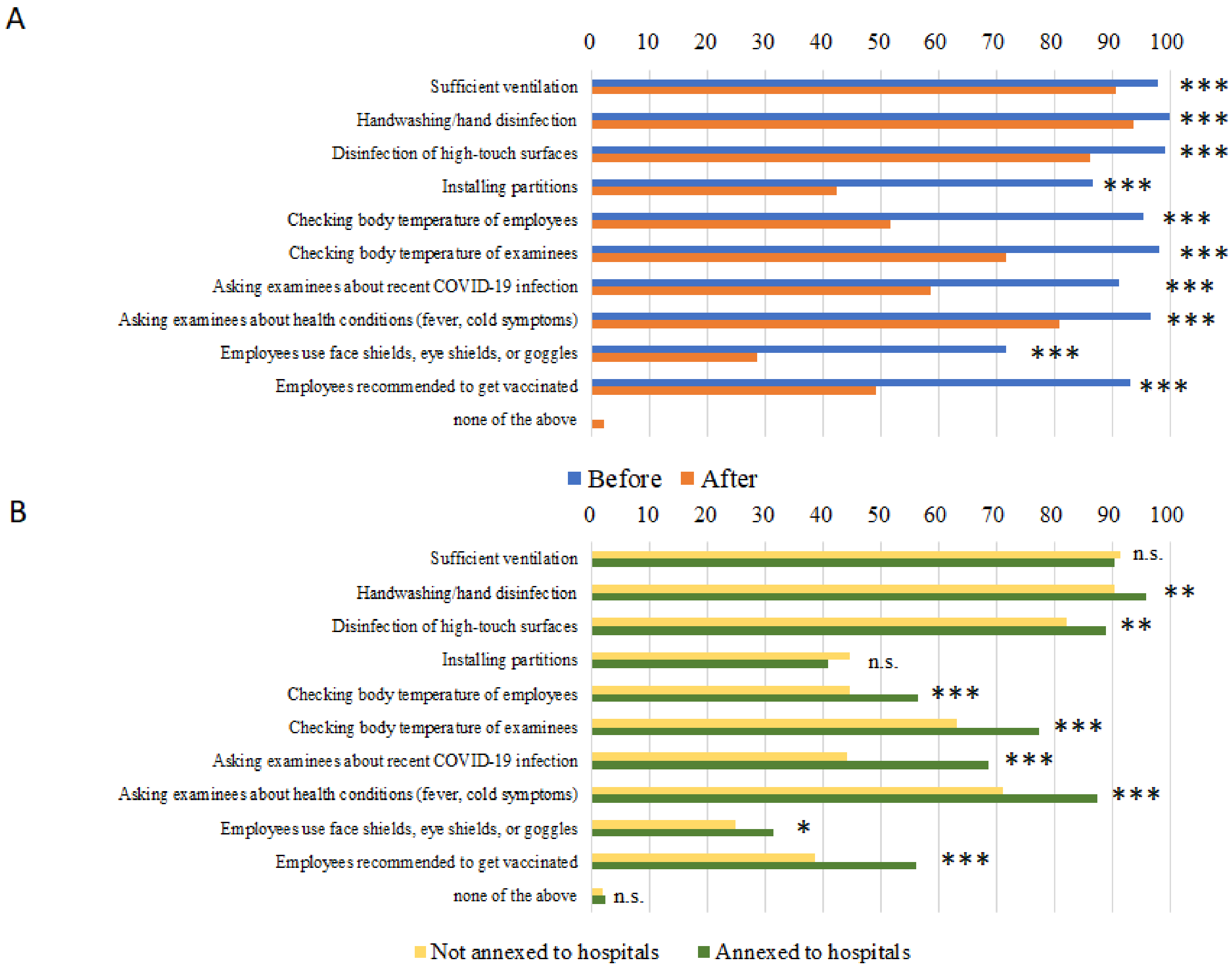

Supplementary Figure 2. (A) Precautions against COVID-19 for performing in-facility check-ups during the year before and the period after the COVID-19 downgrade were compared using McNemar's test (n=959). (B) Precautions against COVID-19 for performing in-facility check-ups after the COVID-19 downgrade were compared between facilities annexed (n=569) and not annexed to hospitals (n=390) using the chi-square test. n.s. not significant, * P <0.05, ** P <0.01, *** P <0.001

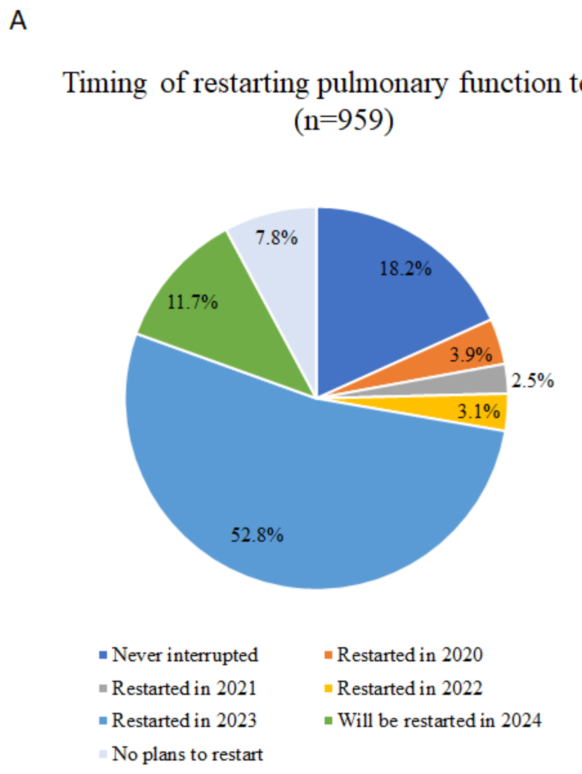

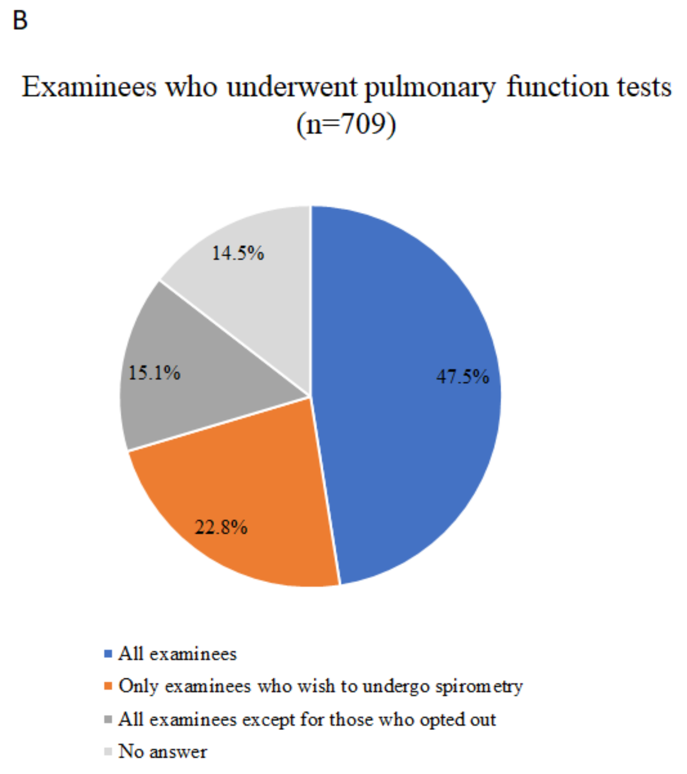

Supplementary Figure 3. (A) Timing of restarting pulmonary function tests (n = 959) (B) Examinees who underwent pulmonary function tests in facilities that restarted/will restart pulmonary function tests (n = 709)
